# Supplementary material for: Identification of drug combinations on the basis of machine learning to maximize anti-aging effects
Source: PLoS One. 2021 Jan 28;16(1):e0246106. doi: 10.1371/journal.pone.0246106 (PMC7843016; doi:10.1371/journal.pone.0246106)
Supplement: S1 Table — (DOCX) [file pone.0246106.s001.docx]

**S1 Table.** Architecture of model

| **Parameter** | **Value** |
| --- | --- |
| # of nodes in input layer | 2 |
| Number of hidden layers | 3 |
| # of nodes in hidden layers | 10, 10, 10 |
| # of nodes in output layer | 2 |
| Learning rate | 0.9532 |
| Learning model | DNN |
| Parameters initialization method | Random_uniform |
| L2 Regularization | 0.001 |
| Activation function of hidden layer | Relu |
| Activation function of output layer | Softmax |
| Number of Epochs | 10 |
