# Supplementary material for: Identification of drug combinations on the basis of machine learning to maximize anti-aging effects
Source: PLoS One. 2021 Jan 28;16(1):e0246106. doi: 10.1371/journal.pone.0246106 (PMC7843016; doi:10.1371/journal.pone.0246106)
Supplement: S2 Table — (DOCX) [file pone.0246106.s002.docx]

**S2 Table.** List of datasets used as training dataset

| **GSE** | **Disease name** | **Age** | **Sex** | **Cell type** | **Number of samples** |
| --- | --- | --- | --- | --- | --- |
| GSE18026 | Chronic Lymphocytic Leukemia | NA | NA | B cell | 7 |
| GSE19147 | B-cell chronic lymphocytic Leukemia | NA | NA | T-cell | 32 |
| GSE22529 | Chronic Lymphocytic Leukemia | >60 years | NA | B cell | 52 |
| GSE28107 | Chronic Lymphocytic Leukemia | 28-69 years | Male  Female | T cells from indolent CLL patients | 6 |
| GSE42733 | Chronic Lymphocytic Leukemia | NA | NA | Purified nurse-like cell | 15 |
| GSE50006 | Chronic Lymphocytic Leukemia | NA | NA | Leukemia cells | 220 |
| GSE28497 | Acute lymphoblastic leukemia | NA | NA | Leukemic cells obtained from bone marrow or blood | 288 |
| GSE42221 | Acute lymphoblastic leukemia | NA | NA | Initial, untreated leukemia | 11 |
| GSE33223 | Acute myeloid Leukemia | 16-88 years | NA | PBMC | 21 |
| GSE42080 | Acute lymphocytic Leukemia | NA | NA | T-cell | 20 |
| GSE32018 | Chronic Lymphocytic Leukemia | NA | NA | Lymph nodes(10 human cell lines) | 30 |
| GSE35179 | Chronic Lymphocytic Leukemia | NA | NA | CD14+ monocytes | 10 |
| GSE5550 | Chronic myelogenous Leukaemia | 24-69 years | Male  Female | Bone marrow CD34+ cell | 17 |
