# Supplementary material for: Identification of drug combinations on the basis of machine learning to maximize anti-aging effects
Source: PLoS One. 2021 Jan 28;16(1):e0246106. doi: 10.1371/journal.pone.0246106 (PMC7843016; doi:10.1371/journal.pone.0246106)
Supplement: S3 Table — (DOCX) [file pone.0246106.s003.docx]

**S3 Table.** Performance of the model

| **Item** | **Results** |
| --- | --- |
| Loss | 0.209075597 |
| Accuracy | 0.953271028 |
| roc_auc | 0.992006346 |
