# Supplementary material for: Identification of drug combinations on the basis of machine learning to maximize anti-aging effects
Source: PLoS One. 2021 Jan 28;16(1):e0246106. doi: 10.1371/journal.pone.0246106 (PMC7843016; doi:10.1371/journal.pone.0246106)
Supplement: S4 Table — (DOCX) [file pone.0246106.s004.docx]

**S4 Table.** Removed genes using DNNs

| **Symbol** | **Full name** |
| --- | --- |
| ZNF37BP | Zinc Finger Protein 37B |
| CFDP1 | Craniofacial development protein 1 |
| SMIM12 | Small Integral Membrane Protein 12 |
| SERPINA3 | Serpin peptidase inhibitor, clade A (alpha-1 antiproteinase, antitrypsin), member 3 |
| COMT | Catechol-O-methyltransferase |
| SLC38A9 | Solute Carrier Family 38 Member 9 |
| ADSS2 | Adenylosuccinate Synthase 2 |
| DGKG | Diacylglycerol kinase, gamma 90kDa |
| AEBP1 | AE binding protein 1 |
| TLE5 | TLE Family Member 5, Transcriptional Modulator |
| DNM1 | Dynamin 1 |
| AGER | Advanced glycosylation end product-specific receptor |
| DYNC1H1 | Dynein, cytoplasmic 1, heavy chain 1 |
| ERF | Ets2 repressor factor |
| CDHR3 | Cadherin Related Family Member 3 |
| FOXL1 | Forkhead box L1 |
| ESYT1 | Extended Synaptotagmin 1 |
| ALOX15B | Arachidonate 15-lipoxygenase, type B |
| FBXO7 | F-box protein 7 |
| FBXO9 | F-box protein 9 |
| AMELY | Amelogenin, Y-linked |
| DISC1 | Disrupted in schizophrenia 1 |
| EIF3K | Eukaryotic Translation Initiation Factor 3 Subunit K |
| GNAS | GNAS complex locus |
| NUDT7 | Nudix (nucleoside diphosphate linked moiety X)-type motif 7 |
