# Supplementary material for: Identification of drug combinations on the basis of machine learning to maximize anti-aging effects
Source: PLoS One. 2021 Jan 28;16(1):e0246106. doi: 10.1371/journal.pone.0246106 (PMC7843016; doi:10.1371/journal.pone.0246106)
Supplement: S5 Table — (DOCX) [file pone.0246106.s005.docx]

| **Rank** | **DNN** | **GEO2R** |
| --- | --- | --- |
| 1 | lycorine | trichostatin A* |
| 2 | vorinostat* | vorinostat* |
| 3 | trichostatin A* | anisomycin* |
| 4 | anisomycin* | lycorine |
| 5 | emetine* | tanespimycin |
| 6 | lanatoside C | emetine* |
| 7 | midecamycin | lanatoside C |
| 8 | cephaeline | digoxigenin |
| 9 | digoxigenin | helveticoside |
| 10 | helveticoside | clindamycin |
| 11 | lobeline | lobeline |
| 12 | tanespimycin | digitoxigenin |
| 13 | proscillaridin | proscillaridin |
| 14 | digitoxigenin | 8-azaguanine |
| 15 | 8-azaguanine | 15-delta prostaglandin J2 |
| 16 | cicloheximide | danazol |
| 17 | luteolin | glibenclamide |
| 18 | apigenin | sanguinarine |
| 19 | clindamycin | chlortetracycline |
| 20 | thiostrepton | alprostadil |

**S5 Table.** Comparison Results of Initial Matching
