# Supplementary material for: Identification of drug combinations on the basis of machine learning to maximize anti-aging effects
Source: PLoS One. 2021 Jan 28;16(1):e0246106. doi: 10.1371/journal.pone.0246106 (PMC7843016; doi:10.1371/journal.pone.0246106)
Supplement: S6 Table — (DOCX) [file pone.0246106.s006.docx]

**S6 Table**. Comparison Results of Secondary Matching

|  | **Vorinostat** | | **Trichostatin** | | **Anisomycin** | |
| --- | --- | --- | --- | --- | --- | --- |
| **Rank** | **DNN** | **GEO2R** | **DNN** | **GEO2R** | **DNN** | **GEO2R** |
| 1 | lycorine | lycorine | lycorine | anisomycin* | vorinostat* | trichostatin A* |
| 2 | anisomycin* | anisomycin* | anisomycin* | lycorine | trichostatin A* | vorinostat* |
| 3 | emetine* | tanespimycin | emetine* | tanespimycin | midecamycin | tanespimycin |
| 4 | lobeline | trichostatin A* | lanatoside C | lanatoside C | lobeline | lanatoside C |
| 5 | lanatoside C | emetine* | digoxigenin | emetine* | lycorine | lycorine |
| 6 | cephaeline | lanatoside C | midecamycin | digoxigenin | tanespimycin | emetine* |
| 7 | midecamycin | digoxigenin | proscillaridin | helveticoside | clindamycin | 15-delta prostaglandin J2 |
| 8 | helveticoside | helveticoside | helveticoside | digitoxigenin | emetine* | clindamycin |
| 9 | digoxigenin | digitoxigenin | lobeline | proscillaridin | danazol | digoxigenin |
| 10 | digitoxigenin | clindamycin | cephaeline | 8-azaguanine | digoxigenin | helveticoside |
| 11 | tanespimycin | 15-delta prostaglandin J2 | digitoxigenin | vorinostat* | glibenclamide | digitoxigenin |
| 12 | proscillaridin | proscillaridin | vorinostat* | sanguinarine | helveticoside | alprostadil |
| 13 | clindamycin | 8-azaguanine | tanespimycin | ouabain | lanatoside C | proscillaridin |
| 14 | 8-azaguanine | alprostadil | 8-azaguanine | clindamycin | luteolin | sanguinarine |
| 15 | trichostatin A* | sanguinarine | apigenin | midecamycin | proscillaridin | lobeline |
| 16 | cicloheximide | ouabain | luteolin | 15-delta prostaglandin J2 | chlortetracycline | danazol |
| 17 | danazol* | pergolide | clindamycin | apigenin | thiostrepton | ouabain |
| 18 | glibenclamide* | cephaeline | cicloheximide | alprostadil | apigenin | midecamycin |
| 19 | apigenin | lobeline | digoxin | lobeline | cephaeline | pergolide |
| 20 | digoxin | midecamycin | thiostrepton | cephaeline | digitoxigenin | 8-azaguanine |
| 21 |  |  | ouabain | strophanthidin |  |  |
| 22 |  |  | danazol* | luteolin |  |  |
| 23 |  |  | glibenclamide* | digoxin |  |  |
| 24 |  |  | chlortetracycline | cicloheximide |  |  |
| 25 |  |  | strophanthidin | verteporfin |  |  |
| 26 |  |  | chlorzoxazone | danazol* |  |  |
| 27 |  |  | ampyrone* | pergolide |  |  |
| 28 |  |  | 15-delta prostaglandin J2 | chlortetracycline |  |  |
| 29 |  |  | baclofen | thiostrepton |  |  |
| 30 |  |  | azacyclonol | geldanamycin |  |  |
