# Supplementary material for: Identification of drug combinations on the basis of machine learning to maximize anti-aging effects
Source: PLoS One. 2021 Jan 28;16(1):e0246106. doi: 10.1371/journal.pone.0246106 (PMC7843016; doi:10.1371/journal.pone.0246106)
Supplement: S7 Table — (DOCX) [file pone.0246106.s007.docx]

**S7 Table**. Neutralized Effect of Combinatorial Drugs

| **TSA_up+Met_down** | | **TSA_down+Met_up** | | **TSA_up+Da_down** | | **TSA_down+Da_up** | |
| --- | --- | --- | --- | --- | --- | --- | --- |
| **Symbol** | **logFC (2nd)** | **Symbol** | **logFC (2nd)** | **Symbol** | **logFC (2nd)** | **Symbol** | **logFC (2nd)** |
| LOC729991 | -0.19178 | C11orf61 | 0.35598 | MAN1A1 | -0.41764 | LPCAT3 | 0.237746 |
| VIPR2 | -0.15803 | C3orf64 | 0.352708 | NEK1 | -0.34197 | HBEGF | 0.196399 |
| TTLL5 | -0.12335 | TRAF2 | 0.341807 | CPM | -0.30355 | SPG21 | 0.190813 |
| MXRA5 | -0.08664 | USP24 | 0.314776 | RPS6KA5 | -0.29529 | SMPDL3B | 0.158208 |
| GRIA3 | -0.08568 | NUP210 | 0.297677 | RTN2 | -0.26521 | VILL | 0.143941 |
| MMP20 | -0.08432 | FAM174B | 0.297142 | ZNF45 | -0.264 | OAS2 | 0.102843 |
| PDLIM3 | -0.07835 | STRN4 | 0.283907 | HEY1 | -0.26307 | ATXN8OS | 0.088654 |
| POF1B | -0.06666 | ERMAP | 0.28357 | GLDC | -0.25948 | ZNF710 | 0.087942 |
| CCR7 | -0.0628 | UBE2L3 | 0.281774 | RUNDC3B | -0.25799 | MED25 | 0.079805 |
| SGK2 | -0.06069 | DNAJB12 | 0.274547 | ZCCHC24 | -0.25237 | TNK1 | 0.073773 |
| **TSA_up+Gli_down** | | **TSA_down+Gli_up** | | **TSA_up+Amp_down** | | **TSA_down+Amp_up** | |
| **Symbol** | **logFC (2nd)** | **Symbol** | **logFC (2nd)** | **Symbol** | **logFC (2nd)** | **Symbol** | **logFC (2nd)** |
| C14orf101 | -0.30378 | IL27RA | 0.238695 | ZNF665 | -0.28665 | PHF15 | 0.172162 |
| SLC25A23 | -0.28536 | POLR3D | 0.228491 | UNC45A | -0.23402 | ZIC1 | 0.014413 |
| NEDD9 | -0.27671 | BIN2 | 0.228167 | PPP2R3A | -0.22732 | CBFA2T3 | 0.035256 |
| GOLGA8A | -0.27653 | CAPN3 | 0.227961 | NEK1 | -0.21916 | PHF2 | 0.028339 |
| C20orf27 | -0.27493 | DMRT1 | 0.210421 | MNS1 | -0.21742 | ZNF692 | 0.039975 |
| SYK | -0.26972 | C12orf35 | 0.205168 | CCPG1 | -0.20877 | ATF5 | 0.028029 |
| VPS39 | -0.26771 | HLA-DQB2 | 0.204459 | PIGL | -0.20792 | P2RY6 | 0.106557 |
| SIPA1L3 | -0.26539 | TMEM39B | 0.204369 | DHRS12 | -0.20211 | PRDM10 | 0.012356 |
| OCEL1 | -0.26432 | FAM160B2 | 0.200078 | RAB23 | -0.20007 | NUAK1 | 0.066955 |
| KCTD2 | -0.26369 | HIST1H4I | 0.198614 | ISL1 | -0.19286 | ARID1A | 0.004172 |
| **TSA_up+Chl_down** | | **TSA_down+Chl_up** | |  |  |  |  |
| **Symbol** | **logFC (2nd)** | **Symbol** | **logFC (2nd)** |  |  |  |  |
| GAN | -0.53626 | KCNK15 | 0.269883 |  |  |  |  |
| PI4K2A | -0.42624 | YSK4 | 0.227081 |  |  |  |  |
| GLCE | -0.39964 | IFT122 | 0.176774 |  |  |  |  |
| TMCO3 | -0.39736 | PRB4 | 0.176718 |  |  |  |  |
| CCDC6 | -0.38829 | ZNF444 | 0.172544 |  |  |  |  |
| H2AFJ | -0.38319 | REL | 0.171224 |  |  |  |  |
| APLP1 | -0.37646 | FUT3 | 0.167033 |  |  |  |  |
| JHDM1D | -0.371 | S100G | 0.163559 |  |  |  |  |
| MYLIP | -0.35572 | MGC12488 | 0.16256 |  |  |  |  |
| SYK | -0.35508 | PADI1 | 0.161449 |  |  |  |  |

| **Ani_up+Met_down** | | **Ani_down+Met_up** | | **Ani_up+TSA_down** | | **Ani_down+TSA_up** | |
| --- | --- | --- | --- | --- | --- | --- | --- |
| **Symbol** | **logFC (2nd)** | **Symbol** | **logFC (2nd)** | **Symbol** | **logFC (2nd)** | **Symbol** | **logFC (2nd)** |
| LOC729991 | -0.19178 | LRRC14 | 0.436859 | EGR3 | -0.86762 | AHNAK2 | 0.817234 |
| INHBA | -0.18101 | SRI | 0.394125 | FJX1 | -0.60519 | ARMCX2 | 0.671376 |
| VIPR2 | -0.15803 | ARHGDIA | 0.364922 | CXCL12 | -0.58611 | CLGN | 0.65048 |
| MYOZ1 | -0.14636 | C19orf40 | 0.3563 | IRX5 | -0.57947 | GLRX | 0.57828 |
| JMJD4 | -0.13692 | SNORA21 | 0.337771 | SLC6A14 | -0.56937 | RPS6KA5 | 0.566006 |
| LLGL1 | -0.12357 | RBL2 | 0.335577 | RASGRP1 | -0.5404 | PCDH9 | 0.54013 |
| TTLL5 | -0.12335 | PACSIN3 | 0.332904 | LY6G5C | -0.52865 | CYP46A1 | 0.528276 |
| MAGEA10 | -0.12023 | MNX1 | 0.330824 | PRDM10 | -0.51782 | TMSB15A | 0.524616 |
| ITGB3 | -0.1167 | UNC93B1 | 0.327871 | NUAK1 | -0.50823 | FYN | 0.512758 |
| CHML | -0.11552 | USP24 | 0.314776 | ZFX | -0.48915 | ANG | 0.504365 |
|  |  |  |  |  |  |  |  |
| **Ani_up+Gli_down** | | **Ani_down+Gli_up** | | **Ani_up+Da_down** | | **Ani_down+Da_up** | |
| **Symbol** | **logFC (2nd)** | **Symbol** | **logFC (2nd)** | **Symbol** | **logFC (2nd)** | **Symbol** | **logFC (2nd)** |
| RHO | -0.36461 | GSTM1 | 0.299524 | CTBS | -0.43846 | SPG21 | 0.190813 |
| MAFG | -0.3278 | TNFSF10 | 0.243481 | ZFP30 | -0.43013 | VILL | 0.143941 |
| C14orf101 | -0.30378 | IL27RA | 0.238695 | FAM59A | -0.42745 | PPT2 | 0.119212 |
| FZD1 | -0.28018 | BIN2 | 0.228167 | ZNF323 | -0.42217 | OAS2 | 0.102843 |
| NEDD9 | -0.27671 | FUT6 | 0.197115 | MAN1A1 | -0.41764 | ZNF710 | 0.087942 |
| URM1 | -0.27013 | POLR2J4 | 0.191004 | KITLG | -0.39699 | MED25 | 0.079805 |
| VPS39 | -0.26771 | LOC100288007 | 0.18737 | HLCS | -0.39546 | TNK1 | 0.073773 |
| EXOG | -0.26667 | ADRA1A | 0.182241 | ATG3 | -0.39424 | ZNF767 | 0.071688 |
| SIPA1L3 | -0.26539 | PRB1 | 0.181311 | MRC2 | -0.393 | RPL10L | 0.058517 |
| KCTD2 | -0.26369 | C16orf57 | 0.177684 | C2orf27A | -0.38783 | LAMA5 | 0.053786 |

| **Vor_up+Met_down** | | **Vor_down+Met_up** | | **Vor_up+Ani_down** | | **Vor_down+Ani_up** | |
| --- | --- | --- | --- | --- | --- | --- | --- |
| **Symbol** | **logFC (2nd)** | **Symbol** | **logFC (2nd)** | **Symbol** | **logFC (2nd)** | **Symbol** | **logFC (2nd)** |
| LOC729991 | -0.19178 | LRRC14 | 0.436859 | SNN | -0.45094 | IL6 | 1.016384 |
| JMJD4 | -0.13692 | SRI | 0.394125 | CCNE1 | -0.40271 | CYP1A1 | 1.00341 |
| TTLL5 | -0.12335 | ZC4H2 | 0.370834 | CAMSAP1 | -0.37338 | KMO | 0.919723 |
| CHRM5 | -0.11252 | ARHGDIA | 0.364922 | LGR4 | -0.37121 | IL24 | 0.86874 |
| MUM1 | -0.08482 | C19orf40 | 0.3563 | ZMAT3 | -0.36417 | NR4A3 | 0.816668 |
| RPS4Y1 | -0.06872 | C11orf61 | 0.35598 | MAT2A | -0.35612 | HIST1H2BC | 0.746231 |
| SGK2 | -0.06069 | KLF6 | 0.355197 | POLQ | -0.35209 | KDM6B | 0.738158 |
| GABRA4 | -0.05865 | C3orf64 | 0.352708 | NCRNA00094 | -0.34524 | CXCL2 | 0.701261 |
| ZBBX | -0.05448 | TRAF2 | 0.341807 | PECI | -0.34033 | LIF | 0.689977 |
| C1orf113 | -0.04878 | SNORA21 | 0.337771 | CROT | -0.32973 | PPP1R15A | 0.687465 |
|  |  |  |  |  |  |  |  |
| **Vor_up+Da_down** | | **Vor_down+Da_up** | | **Vor_up+Gli_down** | | **Vor_down+Gli_up** | |
| **Symbol** | **logFC (2nd)** | **Symbol** | **logFC (2nd)** | **Symbol** | **logFC (2nd)** | **Symbol** | **logFC (2nd)** |
| CTBS | -0.43846 | ZNF710 | 0.087942 | RHO | -0.36461 | TNFSF10 | 0.243481 |
| MAN1A1 | -0.41764 | LAMA5 | 0.053786 | SENP3 | -0.32376 | IL27RA | 0.238695 |
| SERPINA10 | -0.4165 | PHLDA3 | 0.043773 | C14orf101 | -0.30378 | POLR3D | 0.228491 |
| ATG3 | -0.39424 | VPS33A | 0.103886 | SLC25A23 | -0.28536 | BIN2 | 0.228167 |
| MRC2 | -0.393 | SMPDL3B | 0.158208 | FZD1 | -0.28018 | CAPN3 | 0.227961 |
| C2orf27A | -0.38783 | MED25 | 0.079805 | NEDD9 | -0.27671 | PCDHGA10 | 0.216293 |
| PQLC3 | -0.38293 | DTNB | 0.04826 | GOLGA8A | -0.27653 | C12orf35 | 0.205168 |
| C5orf4 | -0.38251 | PPT2 | 0.119212 | KLHDC3 | -0.27629 | HLA-DQB2 | 0.204459 |
| SH2B3 | -0.37197 | LPCAT3 | 0.237746 | C20orf27 | -0.27493 | TMEM39B | 0.204369 |
| TSC2 | -0.37095 | HBEGF | 0.196399 | CHAF1B | -0.26974 | FAM160B2 | 0.200078 |
|  |  |  |  |  |  |  |  |
| **Vor_up+TSA_down** | | **Vor_down+TSA_up** | |  |  |  |  |
| **Symbol** | **logFC (2nd)** | **Symbol** | **logFC (2nd)** |  |  |  |  |
| SERPINB9 | -0.36084 | MAPRE2 | 0.665544 |  |  |  |  |
| ARG2 | -0.33075 | ST3GAL5 | 0.628614 |  |  |  |  |
| ATP8A1 | -0.32557 | LYST | 0.562429 |  |  |  |  |
| EXPH5 | -0.27775 | PCDH9 | 0.54013 |  |  |  |  |
| MAN1A2 | -0.25709 | CYLD | 0.536297 |  |  |  |  |
| AKAP13 | -0.22605 | DNAJC6 | 0.513869 |  |  |  |  |
| GM2A | -0.22451 | PIK3CD | 0.502653 |  |  |  |  |
| PHF20L1 | -0.21521 | FOLR1 | 0.470763 |  |  |  |  |
| LRRC50 | -0.21251 | PDE4DIP | 0.46987 |  |  |  |  |
| STC1 | -0.20434 | JAKMIP2 | 0.454409 |  |  |  |  |
