# Supplementary figures and images for: Identification of drug combinations on the basis of machine learning to maximize anti-aging effects
Source: PLoS One. 2021 Jan 28;16(1):e0246106. doi: 10.1371/journal.pone.0246106 (PMC7843016; doi:10.1371/journal.pone.0246106)

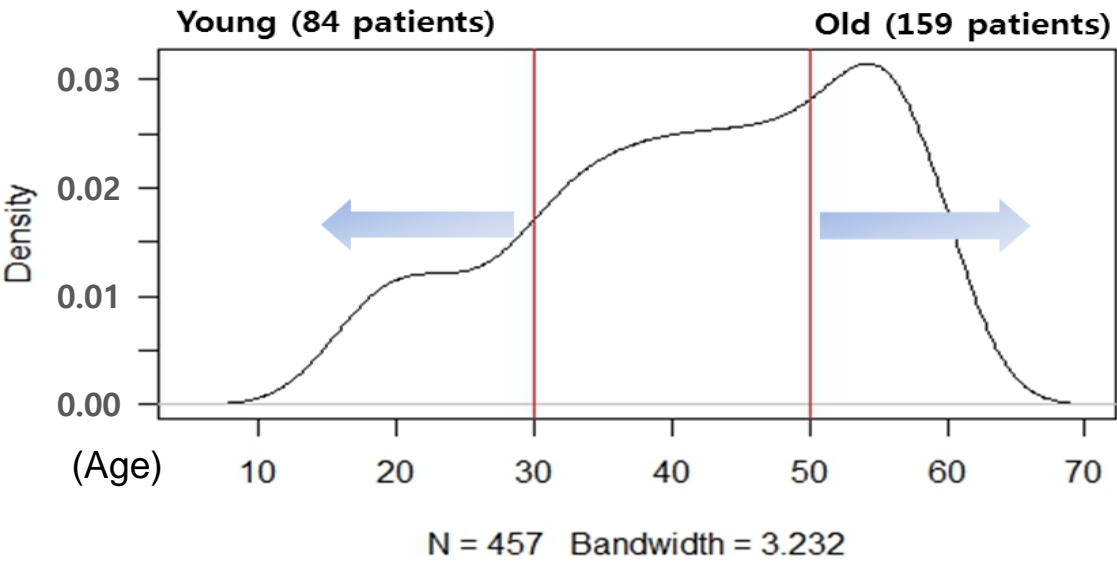

Categorization of target dataset

Supplement: S1 Fig — Categorization of patient groups The samples from patients older than 50 were included in the aged group and younger than 30 were included in normal group. The numbers of samples in the normal and aged groups were 84 and 159, respectively. (PDF) [file pone.0246106.s008.pdf]

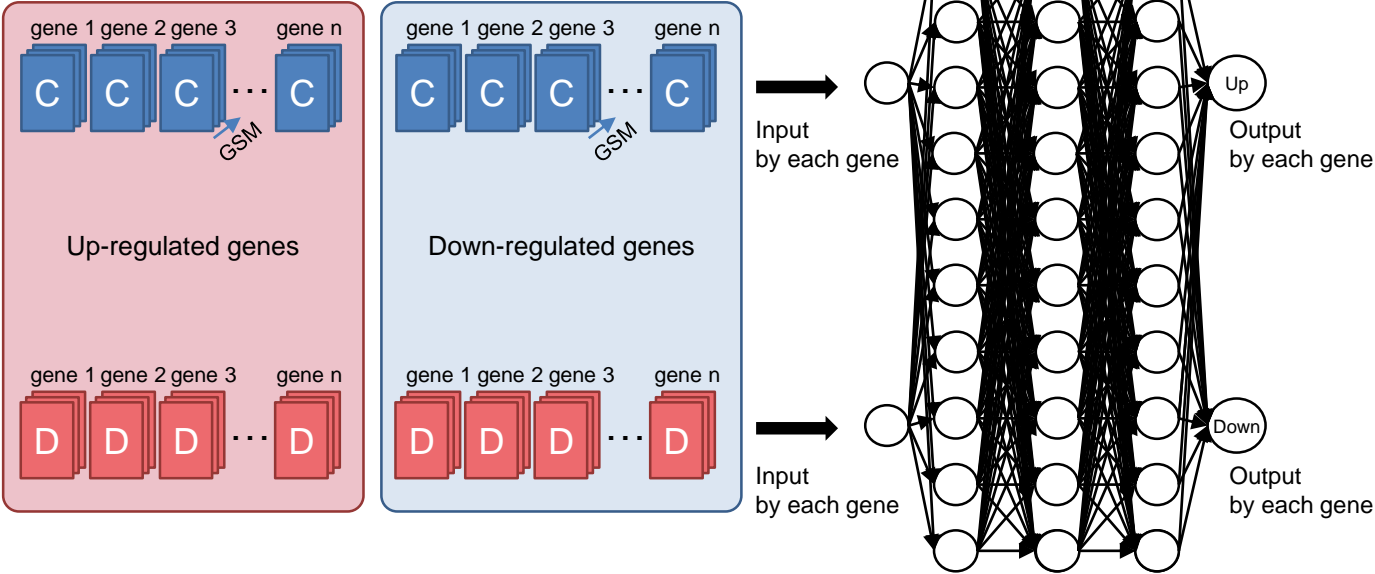

Supplement: S2 Fig — Pairs of samples from Control group (‘C’ in the left side of figure) and Disease group (‘D’ in the left side of figure) are randomly chosen from the same series in the training dataset and fed to the DNN. (PDF) [file pone.0246106.s009.pdf]

Supplementary Figure 3

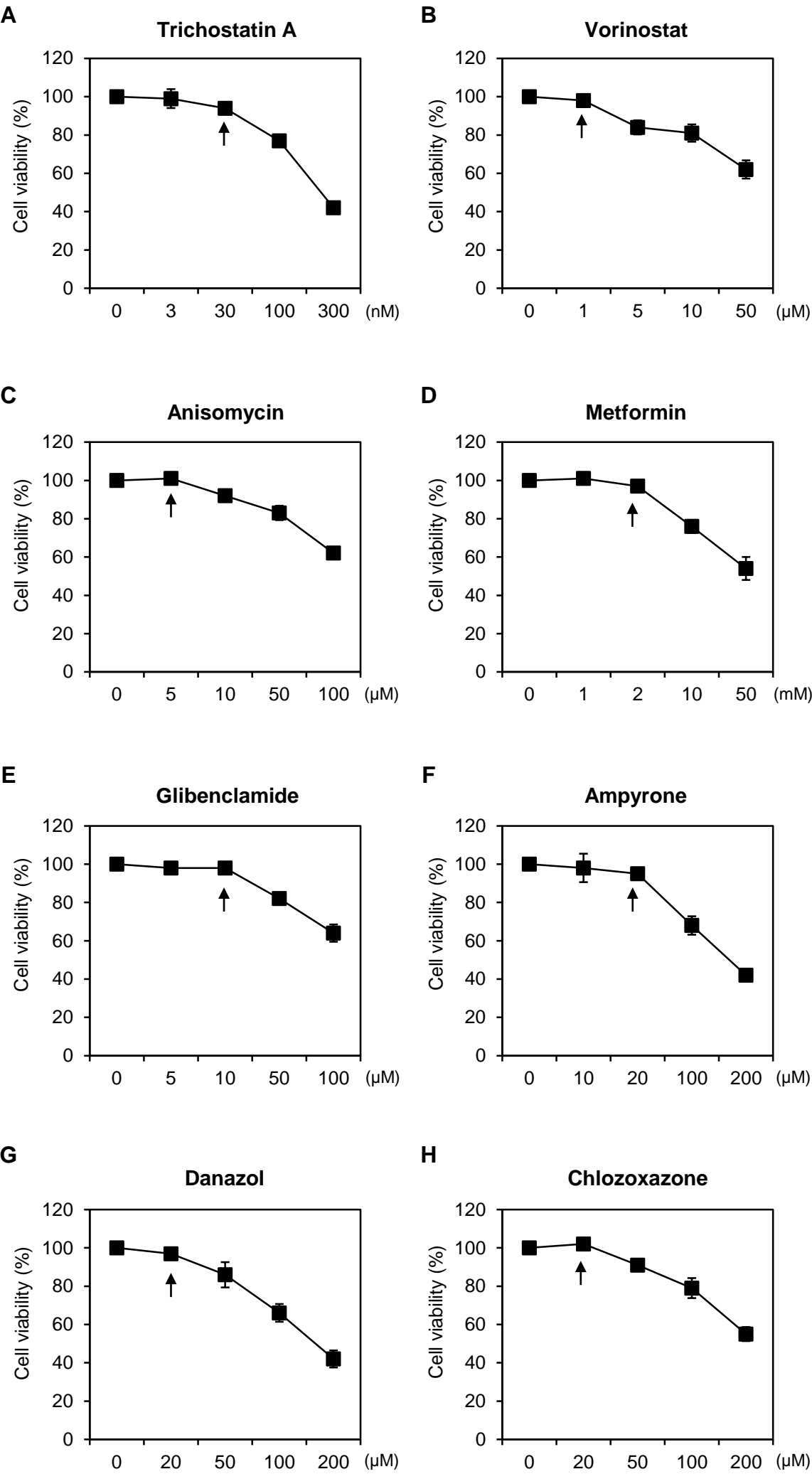

Supplement: S3 Fig — (A) trichostatin A (0–300 nM), (B) vorinostat (0–50 μM), (C) anisomycin (0–100 μM), (D) metformin (0–50 mM), (E) glibenclamide (0–100 μM), (F) ampyrone (0–200 μM), (G) danazol (0–200 μM), and (H) chlorzoxazone (0–200 μM) were treated at the different dosages after 3 hours-pretreatment with 10 μM H2O2 to determine the sub-lethal dose (NOAEL, no observed adverse effects level). (PDF) [file pone.0246106.s010.pdf]

A

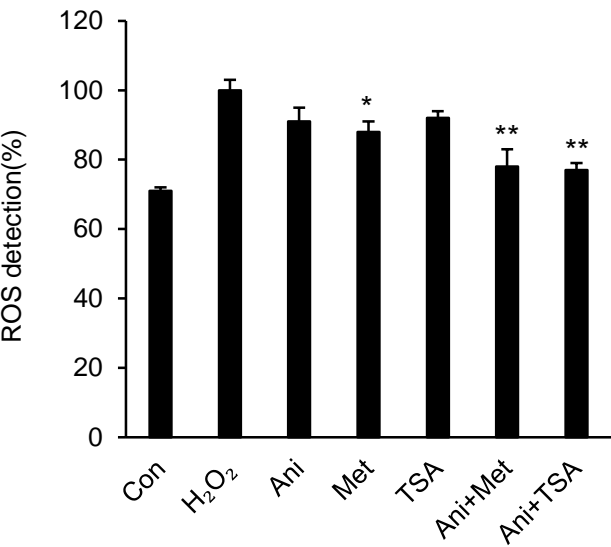

B

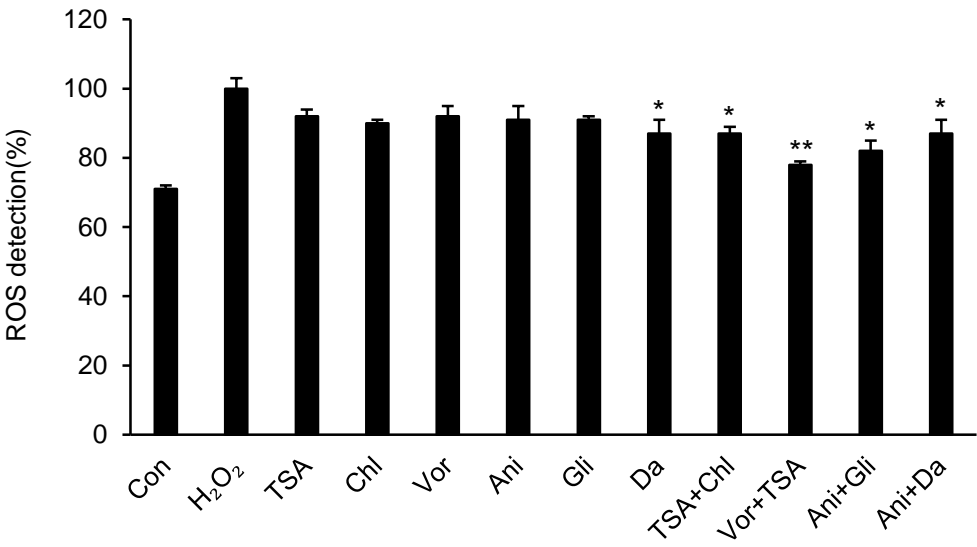

Supplement: S4 Fig — (A) HL-60 cells were co-treated with anisomycin and metformin or trichostatin A for 36 h after incubation with 10 μM H2O2. (B) Cells were also treated with trichostatin A + chlorzoxazone, vorinostat + trichostatin A, anisomycin + glibenclamide, anisomycin + danazol. The intracellular ROS levels were detected by a microplate reader capable of measuring Ex/Em 495/529 nm spectra and recorded. *p <.05, **p <.01 vs H2O2 group. (PDF) [file pone.0246106.s011.pdf]

Supplementary Figure 5

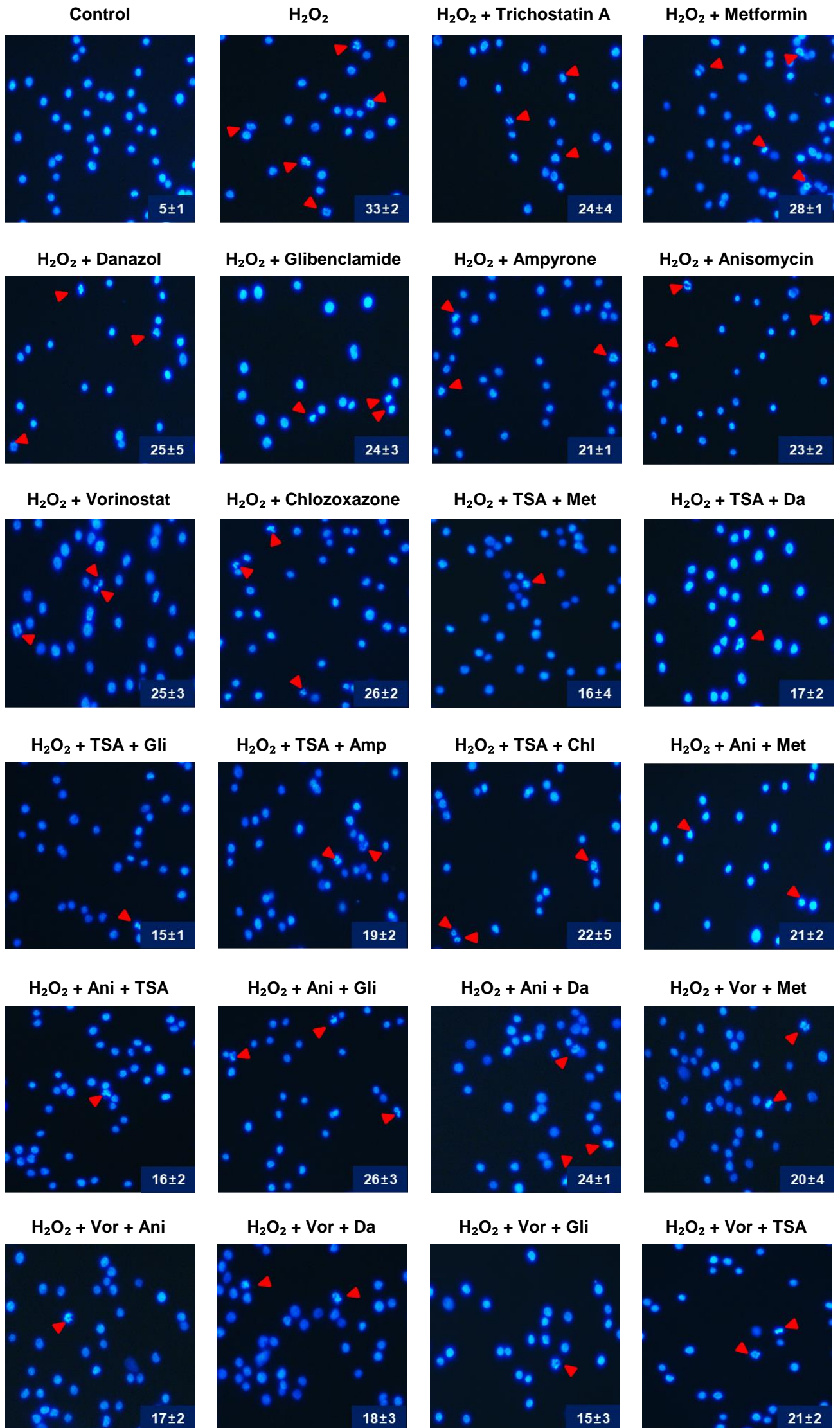

Supplement: S5 Fig — HL-60 cells were treated as indicated in each picture. Nuclei were stained with DAPI, and pictures were taken on a fluorescent inverted microscope. Cells with misshaped(dented) nucleus were indicated by red arrows. (PDF) [file pone.0246106.s012.pdf]

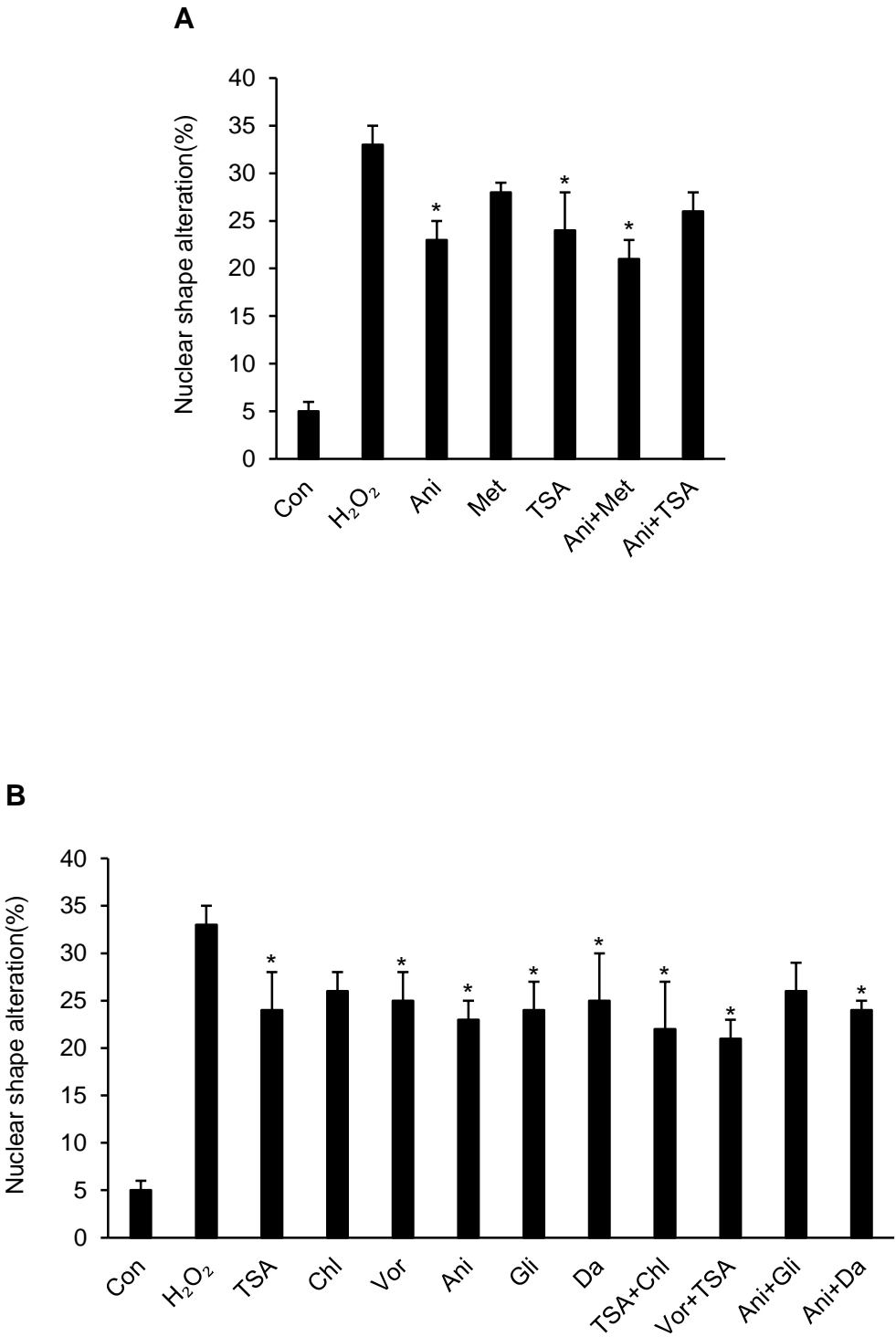

Supplement: S6 Fig — Nuclear morphology changes in DAPI-stained HL-60 cells as shown above were counted and graphed. (A) anisomycin and combined drug pairs, (B) trichostatin A + chlorzoxazone, vorinostat + trichostatin A, anisomycin + glibenclamide, anisomycin + danazol. *p <.05, **p <.01 vs H2O2 group. (PDF) [file pone.0246106.s013.pdf]

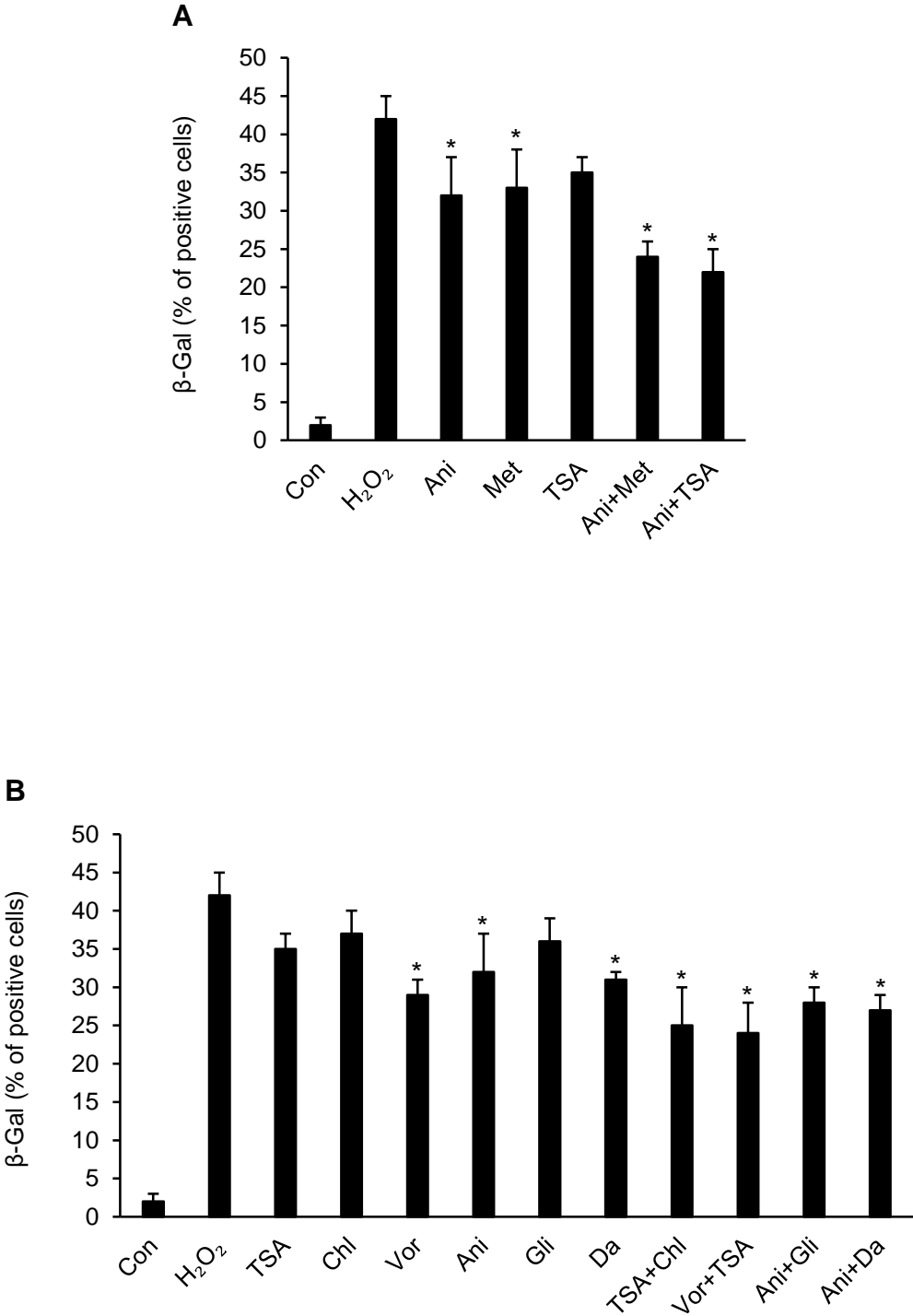

Supplement: S8 Fig — HL-60 cells shown above were quantified and (A) anisomycin and combined drug pairs, (B) trichostatin A + chlorzoxazone, vorinostat + trichostatin A, anisomycin + glibenclamide, anisomycin + danazol. *p <.05, **p <.01 vs H2O2 group. (PDF) [file pone.0246106.s015.pdf]
