# Supplementary material for: Identification of drug combinations on the basis of machine learning to maximize anti-aging effects
Source: PLoS One. 2021 Jan 28;16(1):e0246106. doi: 10.1371/journal.pone.0246106 (PMC7843016; doi:10.1371/journal.pone.0246106)

Supplementary Figure 7

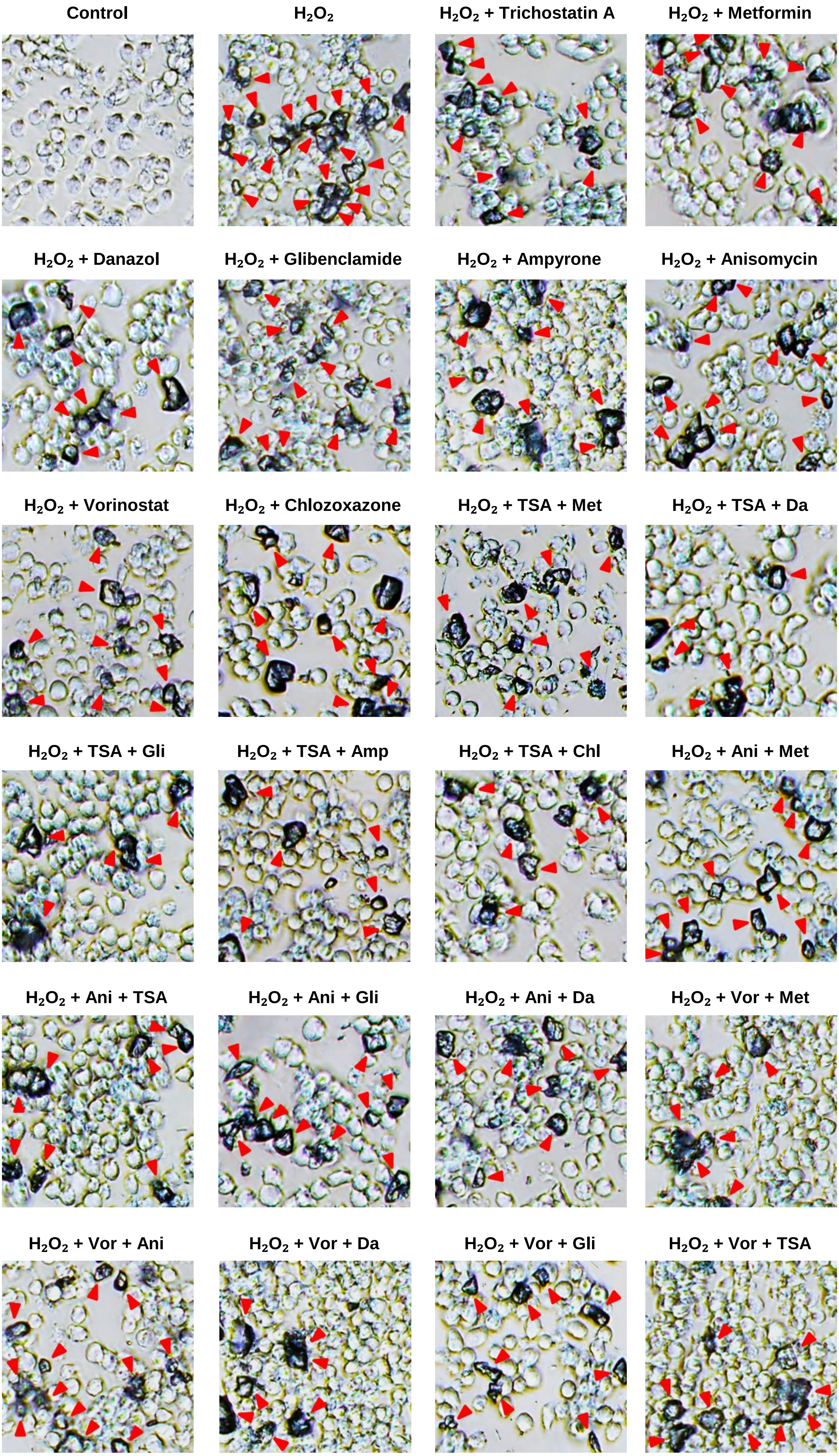

Supplement: S7 Fig — HL-60 cells were treated as indicated in each picture Fluorescent microscopy images were obtained using a fluorescence microscope system, and then beta-galactosidase positive cells were indicated with red arrows. (PDF) [file pone.0246106.s014.pdf]
