# Supplementary material for: Identification of drug combinations on the basis of machine learning to maximize anti-aging effects
Source: PLoS One. 2021 Jan 28;16(1):e0246106. doi: 10.1371/journal.pone.0246106 (PMC7843016; doi:10.1371/journal.pone.0246106)
Supplement: Attachment — Submitted filename: Response to Reviewers.docx [file pone.0246106.s016.docx]

**Reviewer #1:**

**Question 1:** Line 79_duplicated reference ([12] and [15])

**Answer:** We appreciate the kind comment. Since reference 12 and 15 were duplicated, reference 15 was changed to ‘Raghavan R, Hyter S, Pathak HB, Godwin AK, Konecny G, Wang C, et al. Drug discovery using clinical outcome-based Connectivity Mapping: application to ovarian cancer. BMC Genomics. 2016;17(1):811’.

**Question 2:** Line 94_reference [19] “Mephenytoin: a reappraisal. 1976” doesn’t seem to be an artificial neural network paper.
**Answer:** Although the original reference also included this method, we have replaced it with a more specialized one as shown below:

‘Eetemadi A, Tagkopoulos I. Genetic Neural Networks: an artificial neural network architecture for capturing gene expression relationships. Bioinformatics. 2019;35(13):2226-34’.

**Question 3:** Line 96_the sentence is about your current work or about [20]? Generally, I suggest not using references within the description of your current results, because it can be confusing.

**Answer:** As suggested by the reviewer, reference 20 has been removed to avoid any confusion. The sentences in Line 96 do not describe reference 20, but rather describe the results of this paper.

**Question 4:** Line 120_Why did you use a p = 0.1 threshold instead of the regular 0.05?

**Answer:** We understand the reviewer’s concerns. Typically, when identifying DEGs, thresholds are based on *p* <0.05. However, in this study we filtered the gene range of DNN by *p* <0.1 to increase the number of candidates for screening.

**Question 5:** Line 129_I suggest to put both references into the end of the sentence.

**Answer:** As suggested by the reviewer, we have put both references 18 and 22 at the end of the sentence. (Page 6, Line 133)

**Question 6:** Line 134_You altered the words “aging” / “aged” / “old” for the name of the second group through the manuscript. I suggest using a consistence name of the group, e.g “aged group” everywhere.

**Answer:** Following the reviewer’s comment, we amended the names in the main text of all the second groups from ‘old or aging’ to ‘aged group’.

**Question 7:** Line 192 “and selected 1,415 genes” Totally or this is an additional group?

**Answer:** Based on the reviewer’s comment, the text was modified for clarification that it is one cohort as the following: The GEO2R program (https://www.ncbi.nlm.nih.gov/geo/geo2r/) was used to calculate differentially expressed genes between the aged and normal groups of leukemia patients’ cohort (GSE6891), which 1,415 genes had a *p* <0.1. (Page 9, Line 194-196)

**Question 8:** Line 197_I suggest to replace “S1 Table” by the regular “Supplementary Table S1” and use that format in everywhere.

**Answer:** We greatly appreciate and understand the reviewer’s concern, but it was named S1 Table, referring to the submission guidelines of PLOS ONE as the following: ‘Authors may use almost any description as the item name for a supporting information file as long as it contains an “**S**” and number. For example, “S1 Appendix” and “S2 Appendix,” “**S1 Table**” and “**S2 Table**,” and so forth.’

**Question 9:** Line 232_How did you match gene expression data and drugs? I suggest to describe it in the main text too and not only the description of Figure 2.

**Answer:** We acknowledge the reviewer’s comment. To clarify, the text was modified as the following:

We screened common genes between the i-DEG pattern and each d-DEG pattern accordingly and built a common DEG (c-DEG) pattern called initial matching (Fig 2A, 2B). (Page 12, Line 234-236)

Since the drugs found in initial matching cannot reverse all the gene expressions in the o-DEG pattern, a second matching phase was performed to discover other combinatorial drugs to maximize the number of reversed genes with drugs found in initial matching. In the second phase, the genes covered by the chemicals found in initial matching were excluded when scoring the common genes between the i-DEG list and d-DEG patterns. (Page 13, Line 245-250)

**Question 10:** Line 262_Only 91/617 up-regulated genes matched. Isn’t it a too low proportion?

**Answer:** We understand the reviewer’s concern. In our data analysis 91 of 617 genes overlapped (14.7%). Based on computational comparison analysis (5% or more) of overlapping genes is considered valid.

**Question 11:** Line 293_p-value?

**Answer:** Following the reviewer’s suggestion, *p*-value was added for 1^st^ and combination drug treatment. (Page 15, Line 286, 288, 290)

**Question 12:** Line 302_There is no metformin in Table 1 and Table 2. Why did you test it?

**Answer:** We thank the reviewer for the acute observation. Table 1 is DEG patterns using DNNs, Table 2 is i-DEG pattern in initial matching, so metformin data was not added to Table 1 and 2, but added to Table 3, which shows 2^nd^ phase of screening using drug combinations

**Question 13:** Line 323_I suggest to insert a conclusion here like in the previous section. Are the results support the anti-aging effect of the predicted drug-combinations?
**Answer:** We agree with the reviewer’s comment. The conclusion of the nuclear shape alteration result was added to the text.

‘Combination drug treatment restored the nuclear morphology to a spherical shape compared to first drug only treatment.’ (Page 16, Line 319-320)

**Question 14:** Line 346_Conclusion?

**Answer:** As suggested by the reviewer, the conclusion of the senescence-related beta-galactosidase staining results has been added to the text as the following:

‘Taken together, combinational drug treat had a higher percentage of beta-galactosidase compared to single drug.’ (Page 17, Line 343-344)

**Question 15:** Why did you use DNN for determining the differentially expressed genes? It can be calculated directly in more simple ways.

**Answer:** We greatly appreciate reviewer for the comment. We understand the reviewer is curious about why we used DNN rather than the other machine learning systems.

The original version of the expression profiles was modified using a machine learning system called DNN in order to solve the existing problem of the connectivity map where the ‘inserted gene and the drug-derived gene list’, and the ‘target disease and the experimental cell line’ are different. The paragraph was modified for clarification.

‘In silico gene expression pattern-based drug repositioning strategies, such as connectivity map, have been developed as a method for unique drug discovery. However, these strategies have limitations such as lists that differ for input and drug-inducing genes or constraints to compare experimental cell lines to target diseases. To address this issue and improve the prediction success rate, we modified the original version of expression profiles with a stepwise-filtered method. We utilized a machine learning system called deep-neural network (DNN).’ (Page 2, Line 28-34)

**Question 16:** Why did you use leukemia study to train the model? Wouldn’t be better a using only healthy young/old samples for studying aging?
**Answer:** The reviewer raised an interesting point. Originally we intended to isolate PBMC-related factors on aging process. But as you may know, it was too hard to collect the samples or cohort with large number of the properly categorized population. Thus, we decided to utilize leukemia samples excluding the disease factors and isolating the only aging factors. To explain the reason of using leukemia model, the following added to the main text.

‘The purpose of our model was to determine what kind of gene, especially in PBMC (peripheral blood mononuclear cell), was differentially expressed between two groups the young and the old. But as any normal PBMC cohort didn’t meet the enough number of population, we used GSE6891 which was collected for leukemia research including the ‘age’ information.’ (Page 5, Line 93-97)

**Question 17:** What about drug-interference? Does exist a gene that is up-regulated by vorinostat and in the same time downregulated by anisomycin? In that case, anti-aging effect of vorinostats would be neutralized by anisomycin.

**Answer:** We thank the reviewer for the questions. We performed analysis and found genes that were both up-regulated in the first drug and down-regulated in the second drug screening, and vice versa. A total of 14 drug combinations, genes that overlap with Vorinostat and Anisomycin were only valid. Genes down-regulated in Vorinostat were up-regulated with effective values in Anisomycin. (logFC>1, *p*<0.1) However, out of a total of 18,476 genes (down-regulated in Vorinostat and up-regulated in Anisomycin), only two genes IL6 and CYP1A1 were reversely regulated effectively, so it is shown that Anisomycin does not neutralize the effect of Vorinostat. The analysis was added to ‘S7 Table’ and main text. (Page 13, Line 255-263)

**Question 18:** Nobody used these drug-combinations before? Are the experiment are novel results or only reproductions of known effects?

**Answer:** Yes, the drug combinations used in this paper are novel and are the first trial as far as we know.

**Question 19:** Figures are of low quality (hard to read in the PDF). Please use 300dpi.

**Answer:** We appreciate the reviewer’s advice. All figures were changed to 300 DPI.

**Question 20:** Different doses can resulted in different effect. How do you know which dose should be use? Can the method predict appropriate doses?

**Answer:** We appreciate the reviewer’s comments. Even though this a limitation of our technology, the connectivity map we utilized were tried in only a few doses and times. However, based on the statistics, there was range of dose and time calculated. The concentrations that we selected and used were in that range.

**Reviewer #2:**

**Question 1:** Is there any special reason linked to the aging for using the leukemia patients data and cell line in this study? If so, It would be better to briefly describe about it in the introduction section.

**Answer:** The reviewer raised an interesting point. Originally we intended to isolate PBMC-related factors on aging process. But as you may know, it was too hard to collect the samples or cohort with large number of the properly categorized population. Thus, we decided to utilize leukemia samples excluding the disease factors and isolating the only aging factors. To explain the reason of using leukemia model, the following added to the main text.

The purpose of our model was to determine what kind of gene, especially in PBMC (peripheral blood mononuclear cell), was differentially expressed between two groups the young and the old. But as any normal PBMC cohort didn’t meet the enough number of population, we used GSE6891 which was collected for leukemia research including the ‘age’ information. (Page 5, Line 93-97)

**Question 2:** Have you checked any molecular senescence-associated markers like p16 in the combi drug treatment condition?

**Answer:** Following the reviewer’s comment, we performed new experiments to check the level of the markers p16 The results are followed. But the results don’t support in the same line the ROS and beta-galactosidase assay we performed in Fig 5 and 6.

[Only for the reviewers]

The western blot shows that the p16 levels had no significant change upon combination treatment. To explain these results, we searched several publications, which show that p16 may not be the right specific marker for cancer cell lines which already succeeded escaping the senescence. A few convincing papers provides the evidence to support our findings are shown below.

# References:

# Lily I. Huschtscha, et al. p16INK4a and the control of cellular proliferative life span Carcinogenesis, Volume 20, Issue 6, June 1999, Pages 921–926.

G I Shapiro et al. Reciprocal Rb inactivation and p16INK4 expression in primary lung cancers and cell lines, Cancer Res. 1995 Feb 1;55(3):505-9.

**Question 3:** Need to improve the resolution of the Figure 1-3.

**Answer:** We appreciate the reviewer’s advice. All figures were changed to 300 DPI.

**Question 4:** Add the X axis label in the Supple Figure1.

**Answer:** As requested by the reviewer, we labeled the X axis ‘Age’ to S1 Fig.

**Question 5:** What do n-con and con represent in the Figure4B? Need to unify the label format of the control sample between Fig4A and 4B.

**Answer:** The reviewer’s comments are appreciated. As suggested by the reviewer, ‘n-con’ was changed to ‘con’ and ‘con’ was changed to ‘H_2_O_2_’ in Fig 4B.
