# Supplementary material for: Identification of drug combinations on the basis of machine learning to maximize anti-aging effects
Source: PLoS One. 2021 Jan 28;16(1):e0246106. doi: 10.1371/journal.pone.0246106 (PMC7843016; doi:10.1371/journal.pone.0246106)
Supplement: Attachment — Submitted filename: Response to Reviewers.docx [file pone.0246106.s017.docx]

**Reviewer #1:**

**Question 1 (1):** The purpose of the developed DNN is still unclear. Authors use the limma package of GEO2R to find DEGs in leukemia using 13 datasets. Then they developed a machine learning model (based on DNN) that virtually do the same: it can find DEGs in leukemia. To me, the difference is that the input of the DNN is two expression values of a particular gene (one from leukemia patients and one from control patients). Authors used DNN for filtering DEGs of the results of the limma package. If I understand correctly, DNN is used to refine the results of limma. Is it true?

**Answer:** The purpose of our DNN model was to determine if a specific gene was differentially expressed between two samples. In many studies, fold-change approach and statistical methods including t-test and non-parametric test has been used as criterion to select differentially expressed genes (DEGs). But these approaches are sensitive to outliers or sample size. In this study, our goal was to develop a DEGs selection method which is robust to the sample size. The training dataset consists of the DEGs of the results of LIMMA from multiple GSEs with various sample size to this end. (Page 6, Line 116-122)

Overall, I suggest to more written description of the method. In my opinion, in this form, most of the readers (even machine learning and bioinformatics experts) will not understand it. E.g. Please clarify the following questions:

**Question 1 (2):** What is the exact input of the DNN model? Two real number?

**Answer:** Input of the DNN model is a pair of expression value of each gene one from the control group and the other from the disease group.

**Question 1 (3):** What is the exact output? Two real number between 0 and 1? How can you filter by 0.95 if it gives two numbers as an output (Fig S2 shows two output up and down)?

**Answer:** As we described in the article, the output layer has two nodes with softmax as their activation function. Thus, the sum of the output values is 1.0, and we filtered out the value pair if none of the output values are greater than 0.95.

**Question 1 (4):** Machine learning models usually use test sets and not only training set. Please clarify, why do not you need to use a test set?

**Answer:** We divided the dataset into training dataset and validation dataset. The validation dataset was used to evaluate the model and fine-tune the hyper-parameters. We did not create separate test sets in this study because our DNN model was relatively simple to train and we planned to validate the result by experimental method.

**Question 1 (5):** You trained the model using DEGs in leukemia samples compared to control samples. How can it work for drug-induced data that is based on only leukemia samples?

**Answer:** As we mentioned above, the purpose of our DNN model was to determine if a specific gene was differentially expressed between two samples.

**Question 2:** Authors answered to my previous question related DNN as "The original version of the expression profiles was modified using a machine learning system called DNN in order to solve the existing problem of the connectivity map where the ‘inserted gene and the drug-derived gene list’, and the ‘target disease and the experimental cell line’ are different.". It is not clear for me. Why this is a problem? Could you please describe it in more details to readers that are not familiar with CMap (e.g. in the CMap section)?

**Answer:** We use CMap to identify drug candidates to treat particular diseases. CMap can be used to select drugs that can down-regulate the up-regulated genes and vice versa. However, the drug’s gene expression pattern (derived from Cmap) and the inserted gene expression pattern used in the initial analysis do not always match. In other words, the drug derived from CMap does not cover all of the gene expressions for that particular disease.

Second, there is a problem that the cell line used by CMap is different from the disease we want to target. CMap is limited to only five cell lines such as: MCF7 (Breast cancer), PC3 (Prostate cancer), HL60 (Leukemia), ssMCF7 (Breast cancer-charcoal-stripped serum), SKMEL5 (Skin cancer). Therefore, if there are some diseases that we want to target that are not in these five cell lines, it is difficult to derive an accurate drug lists. (Page 4, Line 79-87)

**Question 3:** line 91: Citation [18] doesn't fit here (according to the title of the cited paper). Please check in along with all of the citations.

**Answer:** As suggested by the reviewer, reference 18 has been removed for clarification.

**Question 4:** line 137: "expressiosn ignatures." Please correct the wrong spelling.

**Answer:** Thanks. We corrected it.

**Question 5:** line 198: "Cut-off value" of what?

**Answer:** As the description on the ‘cut-off value’ was overlapped with another former sentence in the same paragraph, the sentence has been removed to avoid a confusion.

**Question 6:** Fig1B/Fig3: what are the labels of the x and y axis?

**Answer:** Originally the number means just the position of the genes in our DEG, with nothing special. It was a simple way to express the compensation (approach to ‘0’) between (+) and (-) genes with ‘white color’ grid.
